# PopCluster Improves Accessibility, Speed and Accuracy of Available Genotypic Clustering Software

**DOI:** 10.1111/1755-0998.70050

**Published:** 2025-09-26

**Authors:** Richard Ian Bailey

**Affiliations:** ^1^ Department of Ecology and Vertebrate Zoology, Faculty of Biology and Environmental Protection University of Lodz Łódź Poland

PopCluster (Wang 2024) represents a significant advancement in population structure analysis software, addressing key computational and methodological challenges that have limited the application of clustering methods to modern genomic datasets. The software, developed by Wang (2024), implements novel likelihood‐based algorithms that substantially improve both speed and accuracy compared to existing methods like STRUCTURE and ADMIXTURE. Its most notable features include memory‐efficient handling of millions of markers and individuals through 2‐bit encoding and distributed computing via MPI, sophisticated treatment of unbalanced sampling through a scaling scheme, and the ability to handle both biallelic and multiallelic markers within a unified framework. PopCluster demonstrates particular strengths when analysing datasets with many assumed populations, weak differentiation between clusters, or highly unbalanced sample sizes—situations where current methods often fail. The software's multi‐platform availability, integrated GUI for Windows users, and built‐in simulation module further enhance its utility for researchers. As genomic datasets continue to grow in size and complexity, PopCluster provides essential capabilities for revealing fine‐scale population structure that would otherwise remain hidden. I discuss the software's innovations in the context of current challenges in molecular ecology and highlight its potential applications in conservation genetics, domestication studies, and understanding complex admixture patterns.

Since its inception, a major focus of population genetics has been on identifying and explaining population structure—the non‐random distribution of genetic variation among individuals and populations. A variety of mechanisms can lead sexually reproducing populations to form two or more distinct multi‐locus genotypic clusters, which may then evolve and adapt independently, leading to further divergence and even speciation, but may also admix and exchange genetic material. Indeed, Mallet (1995) suggested that the maintenance of distinct genotypic clusters in sympatry should be used as a formal definition of species delimitation.

Since the seminal methodological developments of Pritchard et al. (2000) in creating the software Structure, the identification of genotypic clusters and admixture among them from multi‐locus sequence data has become central to a variety of disciplines within the broad framework of molecular ecology. Examples include domestication studies (Matsuoka et al. 2002), human population genetics (1000 Genomes Project Consortium 2015; Allentoft et al. 2024), conservation genetics (Miller et al. 2012), and speciation research (Friedrich et al. 2023). The importance of clustering methods continues to grow as large whole genome datasets become available, allowing highly detailed recovery of clustering and admixture. Historical phylogenomic methods that reconstruct temporal patterns of divergence and subsequent admixture (e.g., TreeMix; Pickrell and Pritchard 2012) are becoming more prevalent, but the original concept of identifying contemporary clusters remains centrally important, not least due to ease of use and interpretation.

With the increasing production of large genomic datasets, improving speed and computational efficiency without sacrificing accuracy has become paramount in the development of new genotypic clustering software. Significant progress has already been made in this direction, including ADMIXTURE (Alexander et al. 2009) and sNMF (Frichot et al. 2014), and the recent addition of the software PopCluster developed by Wang (2024) further increases the speed, accuracy, and accessibility of genotypic clustering and admixture analysis.

One major focus of PopCluster is the efficient use of available memory, both on local computers and distributed clusters, allowing whole genome datasets to be analysed on a laptop, and up to millions of loci from millions of individuals to be analysed on high‐performance clusters. Wang (2024) shows that PopCluster can handle bigger datasets than one of the currently most popular alternatives, ADMIXTURE, and is faster in most circumstances. Another focus is multi‐platform usage, with the software able to run on Windows, Mac and Linux. An available GUI on Windows increases user‐friendliness for users with less experience of building coding pipelines. A further feature that I have personally found useful is the file conversion facility, which can, for example, convert a VCF to a Structure‐style file format.

Major problems occur for clustering software when sample sizes per cluster (typically not known in advance) are small or unbalanced, large k (number of clusters) is assumed, or there are low levels of differentiation among clusters. PopCluster focuses particularly on improved estimation in these circumstances. As shown in Figure 1 of Wang (2024), in extremely bad circumstances, PopCluster dramatically outperforms all other software.

However, not everything can be fully automated, and the user still bears some responsibility in choosing the most appropriate model setup. Wang introduces a “scaling” scheme that allows the user to predetermine how unbalanced their sample is in terms of numbers of individuals per cluster. However, while choosing the correct scaling improves estimation, this is typically not known in advance. The user therefore must use a common‐sense approach to decide whether their chosen scaling value is producing sensible results.

The requirement remains to choose k (number of clusters) in advance, run each k multiple times to deal with stochastic model fitting and a multi‐modal likelihood surface, and run multiple k to statistically compare model fit and decide the appropriate number of clusters. The process can be automated, and with PopCluster, each run is fast, but this nevertheless leads to significant runtime for large datasets.

I would like to add a technical point that is not limited to PopCluster. The original Structure software identified clusters by searching for Hardy–Weinberg and linkage equilibrium, while more recent software does not include this explicit population genetic requirement. As Wang highlights, this means that there is no requirement for loci to be unlinked, and therefore whole genome data can be used. Indeed, in many circumstances, adding more loci increases possibilities to identify true fine‐scale population structure. LD‐pruning remains a common step in many analysis pipelines but is unnecessary from a statistical perspective and, given improvements in computational efficiency, may often also be unnecessary from the perspective of reducing data to a manageable size.

No direct comparison has yet been made between PopCluster and another recent fast clustering software, Neural ADMIXTURE (Dominguez Mantes et al. 2023). However, both provide clear computational improvements over ADMIXTURE. PopCluster is fast, memory‐efficient, multi‐platform, highly accurate, and user‐friendly, making it a welcome addition to the molecular ecology software arsenal.

## Conflicts of Interest

The author declares no conflicts of interest.

## Data Availability

Data sharing not applicable to this article as no datasets were generated or analysed during the current study.
